# The Neurodevelopmental Impact of Neonatal Morphine Administration

**DOI:** 10.3390/brainsci4020321

**Published:** 2014-04-25

**Authors:** Stephanie Attarian, Lan Chi Tran, Aimee Moore, George Stanton, Eric Meyer, Robert P. Moore

**Affiliations:** Division of Pediatric Anesthesiology, Department of Anesthesiology, Washington University in St. Louis, One Children’s Place, Suite 5S-31, St. Louis, MO 63110, USA; E-Mails: Attarian_S@kids.wustl.edu (S.A.); tranl@anest.wustl.edu (L.C.T.); Moore_A@kids.wustl.edu (A.M.); grstant@gmail.com (G.S.); meyere@anest.wustl.edu (E.M.)

**Keywords:** neonate, pain, development, neurotoxicity, morphine

## Abstract

Medical management of newborn infants often necessitates recurrent painful procedures, which may alter nociceptive pathways during a critical developmental period and adversely effect neuropsychological outcomes. To mitigate the effects of repeated painful stimuli, opioid administration for peri-procedural analgesia and ICU (intensive care unit) sedation is common in the NICU (neonatal intensive care unit). A growing body of basic and animal evidence suggests potential long-term harm associated with neonatal opioid therapy. Morphine increases apoptosis in human microglial cells, and animal studies demonstrate long-term changes in behavior, brain function, and spatial recognition memory following morphine exposure. This comprehensive review examines existing preclinical and clinical evidence on the long-term impacts of neonatal pain and opioid therapy.

## 1. Introduction

Intensive care of sick and premature newborn infants includes multiple painful interventions. During the first two weeks of admission to the NICU (neonatal intensive care unit), neonates experience an average of 14 painful procedures per day with the majority occurring during the first few days of life [1]. In light of a growing body of evidence highlighting the detrimental impact of untreated neonatal pain during a critical period in neurologic development, management of this discomfort has become a clinical priority.

Management of neonatal pain often includes opioid therapy. A growing body of evidence suggests long-term harm associated with neonatal opioid exposure. Morphine increases apoptosis in both human microglial cells [2] and neuronal like cells of neonatal rats [3]. Furthermore, animal studies support possible long-term changes in behavior and brain function following the administration of morphine to neonatal rats [4]. Exposure to morphine has been shown to have a strong impact on spatial recognition memory in mice [5]. Although, if administered immediately prior to painful interventions, studies in rats have shown a neuroprotective role for morphine [6].

Clinicians face many dilemmas in the treatment of neonatal pain. Recent studies have demonstrated a reduction in the number painful procedures performed daily [7]. However, this reduction was modest and many painful procedures still exist. Despite this, many physicians remain reluctant to administer morphine as they fear detrimental neurological effects and are unsure of it’s role after recommendations from Bellu’s 2008 Cochrane Review [8]. The pressure clinicians face when managing this fine balance remains a topic of discussion in neonatal intensive care units worldwide. This comprehensive review highlights the need for analgesic therapy and examines existing preclinical and clinical evidence on the long-term impacts of neonatal opiate therapy.

## 2. Adverse Effects of Neonatal Pain

### 2.1. Early Development of Pain Pathways

Intensive care facilitating survival of infants born at younger gestational ages often entails lifesaving but painful procedures, yet the long-term developmental impact of repeated exposure to noxious stimuli in early life remains poorly understood. Sensory receptors appear early in fetal life [9], with an integrated nociceptive pathway present at 24–28 weeks’ gestation [10]. Although neonates were thought to have high pain thresholds as an adaptive mechanism to tolerate the birthing process [11], this understanding has been revised in light of studies demonstrating clear response to noxious stimuli at the spinal and brainstem reflex level in extremely premature neonates [12]. More recent studies using near infrared spectroscopy monitoring have shown cortical responses to noxious stimuli in neonates as young as 25 weeks post conceptual age, suggesting pain response integrated above the brainstem-reflex level [13]. Since these nociceptive pathways present in hospitalized premature infants are repetitively stimulated during a critical neurodevelopmental period [14], it is important to begin to understand the short and long term neurological effects of painful stimuli.

### 2.2. Impact of Neonatal Pain

Published data are on the effects of neonatal pain are limited due to a variety of challenges. First, it is difficult to consistently and objectively measure the effects of painful procedures. Most pain assessment tools rely on both objective measures (e.g., tachycardia) and behavioral cues (e.g., crying). Vital signs can be affected by other factors such as drug administration, body temperature, and intravascular volume status; it can be difficult to discern in a critically ill infant whether a change in vital signs is attributable to discomfort or to the infant’s comorbidities. Behavioral changes are affected by postmenstrual age and temperament, and behavioral responses in preterm infants may be blunted and delayed relative to their term counterparts [15]. Very few pain assessment tools are adjusted for post conceptual age and none of them consider repetitive painful events [12].

Infants born prematurely are at risk for neurodevelopmental delay and disability. While poor neurologic outcomes are likely multifactorial in etiology, the potential for painful stimuli in early life to alter neurologic development remains incompletely understood. For the first two weeks of admission to the NICU, neonates experience an average of 14 painful procedures per day with the majority occurring during the first few days of life [1]. Acute pain may precipitate a stress response with adverse physiologic consequences, such as impaired ventilation, changes in intrathoracic or arterial pressure, and vasoconstriction of vital organs [16,17]. Such acute physiologic changes may contribute to the pathogenesis of intraventricular hemorrhage or periventricular leukomalacia in at-risk infants [18]. Painful events can affect complex behavioral responses for up to 24 h after the event; altered sleep-wake cycles, feeding, and crying have been documented in male infants circumcised without local anesthetic [11].

Understanding alterations in sensory processing associated with pain is dependent on both the method of evaluation and the age of the child [19]. Despite these limitations, there have been a number of studies suggesting increased behavioral responses to subsequent painful procedures if the child has experienced prior painful procedures [13]. Full term male neonates who were circumcised had increased behavioral responses during subsequent vaccinations compared to their uncircumcised counterparts [20]. While analgesic treatment with sucrose may dampen the effects of the acute pain in neonates with repeated skin break occurrences, it does not decrease remote hyperalgesia [21]. This suggests that pain during this crucial period of development may lead to neurologic remodeling [12,20].

Alteration of neuronal responsiveness may explain why repeated exposure to noxious stimuli may alter development of pain perception, leading to hypersensitivity and chronic pain. Although integrated nociceptive pathways are functional by 24–28 weeks gestation, systems modulating pain remain immature [13]. For premature infants, the development of these modulatory pathways coincides with the highest frequency of noxious stimuli are performed, leading to long-term increased sensitivity to pain [22]. Skin over wounded areas shows increased afferent fibers and hypersensitivity of neurons which is hypothesized to effect long-term changes in the spinal cord, as was found in a preclinical model [23,24]. This hypersensitivity may contribute to preterm neonates requiring higher levels of anesthetics and analgesics to achieve sedation than their term counterparts [16]. This increased sensitivity combined with a lack of mature inhibitory mechanisms, leads to the windup phenomenon, with neurons gaining increasing excitability with each painful stimulus. To neonates experiencing the windup phenomenon, even routine care such as bathing and diaper changing can be noxious [25]. The inability to modulate recovery wanes around 32 weeks postmenstrual age [26,27]. Development of hypersensitivity may be altered by analgesia: a study of infants with chronic pain due to repeated skin break procedures performed on the same foot showed that hypersensitivity to subsequent heel lances was mitigated with local anesthetic cream [26]. Furthermore, brain MRIs (magnetic resonance imaging) of preterm neonates exposed to repeated stimuli quantified by the number of skin break procedures showed inhibited brain maturation and development [28]. It remains a challenge to assess pain and provide analgesia for such frequent and intermittent stimuli.

Surgery is an obvious source of pain for some neonates, and several studies have focused on how analgesia can mitigate the short- and long-term consequences of postoperative pain. Although it is difficult to assess neonatal perception of pain, postoperative morbidity and mortality are useful endpoints for effective control of postoperative pain. Anand [11] first demonstrated the impact of analgesia on postoperative morbidity and mortality in a randomized controlled trial of preterm neonates undergoing patent ductus arteriosus ligation. Infants given fentanyl had significantly decreased stress hormones (epinephrine, norepinephrine, glucagon, aldosterone, corticosterone, and other steroid hormones and fewer postoperative complications [29]. The potential of operative pain to precipitate hyperalgesia or an altered pain threshold may be mitigated by adequate analgesia: studies have shown that infants subjected to surgery with perioperative morphine for analgesia show a similar behavioral response to vaccinations as their peers who were never subjected to surgery [19].

Parents play an important role in helping providers assess pain in older children and in those neonates who have been stable enough for discharge home. When parents of former preterm infants were surveyed at 18 months corrected age, they reported lower sensitivity to pain of everyday bumps and bruises. A study of pain response at the eight-month follow-up visit showed that despite ex-preterm infants having hypersensitivity to pin prick, they had faster recovery times than their term counterparts [25]. Interestingly, parents of former preterm infants, report more functional pain and somatization complaints when the children are school age. This seems be correlated with length of NICU stay and exacerbated if maternal involvement and responsivity is low. Although not the only factor, the prevalence of painful procedures during NICU may contribute to this later somatization [19,30]. As these children age, they do not seem to report more pain than their healthy counterparts, but their pain is more likely to interfere with activity [19].

Although it is difficult to control for the effects of comorbidities (e.g., mechanical ventilation, infection, steroid exposure), it is increasingly clear that pain-related stress can produce long-term changes in the developing brain. Increased numbers of skin-breaking procedures have been recently associated with lower scores on mental and psychomotor indices [31]. Functional brain MRIs of former preterm neonates showed increased activation of sensory areas in response to pain compared to former full term, non-hospitalized controls [32]. Pain experienced before 32 weeks post conceptual age has been identified as a factor in growth impairment, as well as reduced white and gray matter maturation [15]. Pain-related stress has been correlated with cortical thinning at seven to eight years of age in preterm children without any history of severe brain injury in the neonatal period and without significant cognitive, sensory, or motor impairments [33]. Repetitive episodes of pain may also contribute to neuronal damage linked to abnormal behavioral regulation later in life manifesting as attention deficit/hyperactivity disorder, separation anxiety disorder, and phobias [18,23]. In aggregate, these results are highly suggestive of the deleterious long-term neurodevelopmental effect of neonatal pain.

### 2.3. Consensus Views Support Analgesia and Limitation of Painful Procedures

Despite repeated evidence that pain is detrimental to neonates, recent studies have demonstrated that only one third of patients receive preemptive analgesic therapy [1]. Quantifying neonatal pain has challenges, but pain management is an important part of maximizing the outcome of the sick neonate. Neonates can develop chronic pain, and become sensitized to even the gentlest stimulation via the wind-up phenomenon [34]. The 2001 Consensus Statement for the Prevention and Management of Pain in the Newborn has guidelines for recognition of pain as well as treatment protocols and drug dosing for common neonatal procedures. Minimizing frequent procedures is the most obvious way to decrease neonatal pain followed by non-pharmacologic interventions such as swaddling and nonnutritive sucking. If these are unsuccessful, and the intervention or procedure remains necessary, judicious use of analgesic medications should be applied.

## 3. Morphine’s Impact on Neuronal Apoptosis

While there is mounting evidence supporting the benefits of analgesia for newborns and pre-term infants, opioids have been shown in preclinical and clinical studies to influence neuronal cell death. Multiple opioid receptor agonists have been investigated on their roll in apoptosis including morphine. Researches have utilized multiple assays for assessing morphine’s affect on apoptosis in numerous types of cells including microglia and neurons. Specifically, Hu *et al.* [2] investigated the impact of morphine *in vitro* on fetal microglia, (which, like many immunocytes, express opioid receptors), astrocytes, and neurons, using caspase-3 expression as a marker for apoptosis. Caspase-3, part of a family of intracellular cysteine proteases, has been directly linked to apoptosis. Microglia, neurons, and astrocytes, were exposed to control, low dose (10^−6^ M) or high dose (10^−4^ M) morphine for five days. Their results demonstrated a significant increase in apoptosis of morphine-exposed microglia and neurons via a caspase-3 dependent pathway. Astrocytes did not show this relationship. Apoptosis of morphine-exposed microglia and neurons were reversed by the coadministration of naloxone, strengthening the argument that this is an opioid-mediated phenomenon.

While Hu, *et al.* [2] explored the effects of morphine on human cells, much of the data has been born out of the pre-clinical model. For example, the apoptotic effect of morphine has also been explored in rat models. Boronat *et al.* [35] devised a studied the effects of morphine on the immunodensities of the pro-apoptotic Fas receptor and anti-apoptotic Bcl-2 oncoprotein rat brains. Male rats were divided into an acute (one-time dosing) or chronic (three times a day for five days, or twice a day for 13 days) morphine administration regimens. Other groups were also treated with naloxone in addition to morphine. Brain tissue was collected following sacrifice of the animals, and FAS and Bcl-2 proteins were measured by immunoblotting and immunoreactivity techniques. The pro-apoptotic Fas receptor was significantly upregulated and the anti-apoptotic Bcl-2 receptor moderately downregulated in the chronic morphine group. These findings were not present in the acute morphine and naloxone-treated mice, suggesting this protein regulation may be mediated via the activation of opioid receptors.

Katebi *et al.* [36] explored the effects of morphine on pro-apoptotic (Caspase-3, Bax) and anti-apoptotic (Bcl-2) proteins in the nucleus accumbens (NAc) and prefrontal cortex of rats. They dosed their rats with morphine three times a day for five days, with several different concentrations: 0.5 mg/kg, 5 mg/kg, or 10 mg/kg. Apoptotic proteins from these anatomical areas were detected using immunoreactive polypeptides and quantified by Western blot. Bax/Bcl-2 ratio was increased compared to control in all morphine doses, and in a reverse dose-dependent manner in the nucleus accumbens. In addition, the level of cleaved caspase-3 in the NAc increased significantly in the 0.5 mg/kg morphine treated group, more so than the 5 or 10 mg/kg treated animals. These data indicate, by using specific apoptotic protein densities, that morphine administered at varying doses over several days increases protein involved in apoptosis in the NAc and prefrontal cortex.

Given the aforementioned studies, it can be concluded that there is some evidence of morphine altering apoptotic protein expression in various regions of both human and rat brain tissue. However, it should be noted that in all of these animal models, morphine was administered in the absence of noxious stimuli, which provides an incomplete model of analgesia in the NICU and cannot assess the adverse effects of opioid administration weighed against the adverse effects of pain. Extrapolation of data from the preclinical model to human neonates is also complicated by the mature age of the animals, as well as the large doses of morphine used. Adult animal brains are likely to demonstrate less plasticity and recovery while large morphine doses may lead to additional injury by other factors such as hypotension and hypoventilation. In the realm of pediatric anesthesia, it is of vital importance to understand the clinical context, benefits, and potential adverse effects of all medications administered. Although these data do not directly test neuronal apoptosis in human children exposed to opioids, they at least suggest further investigation into what signaling pathways opioids are involved with in addition to pain pathways.

## 4. Neuroprotective Impact of Analgesia

The studies of murine and human fetal models discussed above showed that opiate-toxicity may inhibit growth, promote apoptosis, and impair regeneration of glial cells. Based on these studies, there are mounting concerns regarding the use of opiates in human neonates. This is a conundrum particularly in the neonatal ICU, where opiates are utilized to ameliorate pain and decrease agitation, which also have deleterious effects on early neurodevelopment. To adequately assess the impact of opioid therapy on outcomes of sick neonates, a model that takes into account the competing adverse effects of pain and opioid therapy is required.

It is well known that pain stimuli reach the cortex, induce pain-specific activation, and alter peripheral and central pain processing in neonates. Repetitive neonatal pain in rats accentuates neuronal excitation and increased cell death in several cortical and subcortical areas, suggesting that pain may have widespread effects on brain development with short- and long-term ramifications. Neonatal rats subjected to repetitive painful injections showed histological evidence of increased brain cell death in the frontal and parietal cortex compared to untreated rats. These effects were exacerbated by increased pain intensity: neonatal rats exposed to severe, inflammatory-type pain had significantly higher brain-cell apoptosis scores and altered expression of proteins involved in early thalamic and cortical development compared to rats exposed to mild pain [37]. Earlier studies have demonstrated the effects of pain severity on gene expression in the neonatal mouse hippocampus. Mild stress led to decreased expression of genes related to cell maintenance, while severe stress lead to increased expression of gene sets related to neurodevelopment and inflammation, and decreased expression of gene sets responsible for cellular repair [38]. Collectively, these results suggest a dose-dependent correlation between pain intensity and brain-cell apoptosis.

Although pain and environmental stressors clearly lead to altered neurodevelopment, assessing the combined effects of stress and opioids on neurodevelopment is complex. Emerging evidence suggests that morphine may have protective effects against nerve cell apoptosis under conditions of pain and stress [6]. Both neonatal morphine exposure and repetitive pain have been shown to cause behavioral changes and abnormal learning in rats; these effects were reduced when pain and morphine treatment were combined [39]. On the molecular level, morphine has been shown to down-regulate stress-related changes in gene expression, protecting cells against apoptosis [40]. Morphine also alters the expression of protein kinase C epsilon (PKCvarepsilon), a protein involved in stabilization of synaptic connection, and doublecortin (DCX), which is involved in neuronal migration and differentiation in the cortex [41,42]. In the setting of pain, PKCvarepsilon and DCX are shown to be abnormally overexpressed, which may lead to NMDA-receptor mediated excitotoxic cell death, and aberrant neuroblast differentiation and migration [42,43,44]. However, pretreatment with morphine prior to pain exposure in neonatal rats lead to decreased expression of PKCvarepsilon and DCX. These studies suggest a neuroprotective role of morphine administration for the pretreatment of pain.

Although pre-emptive analgesia appears to be neuroprotective, the modulatory effect is limited to specific situations. In neonatal mouse models, morphine exposure had different effects on hippocampal gene expression depending on the severity of environmental stress. Treatment of mild stress with morphine led to increased expression of specific gene sets related to neurodevelopment. In contrast, treatment of severe stress with morphine down-regulated genes involved in RNA processing and neurodevelopmental processes, which could be a mechanism by which morphine leads to decreased brain growth [38]. Potential neuroprotective effects of morphine are influenced by both the severity and the duration of stress, complicating the assessment of benefits of prolonged opioid pharmacotherapy for recurrent noxious stimuli.

Many studies have shown deleterious neuromodulatory effects of opiates, but upcoming research has demonstrated that morphine may have protective effects in specific conditions of pain and stress. Therefore, opioid pharmacotherapy may have an appropriate role in the NICU if appropriately tailored to individual babies who have pain. It remains unclear how long human neonates can safely be exposed to opiates or whether morphine has any significant neuroprotective effects in human neonates. Limited data in human preterm neonates suggests adverse effects from prolonged preemptive exposure to analgesia.

## 5. Long-Term Impact of Morphine Therapy-Clinical Evidence

There is a growing body of clinical literature on long-term neurodevelopmental outcomes of neonatal morphine administration. Several recent studies have examined large cohorts and employed well-established outcome measures. Three studies are summarized below in Section 5.1, Section 5.2 and Section 5.3.

### 5.1. A pilot Study of Preemptive Morphine Analgesia in Preterm Neonates

Effects on Head Circumference, Social Behavior, and Response Latencies in Early Childhood [45]. This study reports growth and neurodevelopment outcomes of 14 morphine-treated infants (eight male and six female) and five placebo-treated infants (four male and one female) who were a part of the NEOPAIN trail. The NEOPAIN study examined the impact of preemptive morphine for treating the discomfort of premature infants between 23 and 32 weeks gestational age who required intubation in the first 72 h of life. In the original study, 212 infants were randomized to receive preemptive morphine or placebo within the first 8 h following intubation and continued for a maximum of 14 days [46]. Open-label morphine use was permitted in either group, with additional guidelines for administration of phenobarbital, opiate antagonists, and muscle relaxants as clinically indicated. Midazolam and other sedatives were excluded from both groups. Results of this subgroup follow-up are outlined below.

Somatic growth parameters showed no difference between the groups in height or BMI, but significantly lower weight (effect size 0.81) and head circumference (effect size 2.81) in the morphine-exposed group. One potential explanation for this significant decrease in weight and head circumference measures is the delay in reaching full enteral feeds in morphine *vs.* placebo-treated infants in the original NEOPAIN trial. Notably, early growth in the NICU has been more predictive of developmental outcomes at 5 years of age than growth in the first years following discharge [47].

Neuropsychological outcomes were assessed using standardized tests (Stanford-Binet IQ testing, Wide-Range Achievement Test/WRAT4) and parent questionnaires (Child Behavior Checklist, Vineland Adaptive Behavior Scale, Conners’ Comprehensive Behavior Rating Scale). Although there were no significant differences between morphine- and placebo-treated children in Stanford-Binet IQ scores or academic achievement as measured by the WRAT4, parental surveys suggested increased social problems in morphine-treated infants. The Conners’ Comprehensive Behavior Rating Scales showed a statistically significant increase in social problems, specifically in the areas of creating and maintaining peer relationships, as rated by parents in the morphine treated group. The Vineland Adaptive Behavior Scale also showed some adverse effects of morphine, but is of limited significance as scores could only be computed for one placebo-treated child.

Operant testing was also conducted, in which progressive ratio (PR) task measuring the number of reinforcers earned and delayed matching to sample (DMTS) tasks using press plates and levers to match images and gauge latency and response times were performed. There were no statistically significant differences between the groups in the PR task. However, DMTS testing showed a significant negative effect on task completion in morphine-treated infants (effect size 0.96) and increased choice response latencies (general and correct choice) in the morphine group. Incorrect choice latency did not differ between placebo and morphine treated groups.

This small study showed no difference in IQ, self-sufficiency, motivation or school-related performance between the placebo and morphine treated groups. These results are consistent with prior studies showing that preemptive morphine administration does not cause significant IQ or academic performance alterations [48,49]. However, this study did raise some concerns regarding subtler long-term harms of opioid administration by looking including increased parent-reported social problems, increased response latencies, lower task completion rates, lower body weights, and smaller head circumferences in the morphine-treated group.

### 5.2. The Second Study Entitled

“Does Neonatal Morphine Use Affect Neuropsychological Outcomes at 8 to 9 Years of Age?” [50] is a long-term follow-up that drew subjects from a cohort of 150 mechanically ventilated infants ≤ 3 days of age who were randomized to receive continuous morphine (*n* = 73) or placebo (*n* = 77), with open-label morphine used for children with clinical evidence of pain [51]. In a follow-up study occurring at 5 years of age [52], no significant differences were found between morphine-treated (*n* = 49) and placebo-treated (*n* = 41) infants in visual-motor integration (Beery VMI score), behavior (parent-completed Child Behavior Checklist and teacher-completed Teacher Report Form), chronic pain, or health related quality of life (Health Utility Index). Overall IQ scores were slightly but significantly lower in the morphine-treated group, however the negative impact of preemptive morphine treatment vanished when the regression model was revised to adjust for the propensity score and to include open-label morphine therapy and primary ventilation as explanatory variables. In contrast with overall IQ scores, the visual analysis subtest of the IQ test was significantly lower in the preemptive-morphine group, even in multivariate models.

The current study follows up the same children at 8–9 years of age with a goal of extensively assessing the effects of morphine on executive function (Cambridge Neuropsychological Test Automated Battery/CANTAB, Behavior Rating Inventory of Executive Functioning/BRIEF questionnaire), intelligence (Wechsler Intelligence Scale for Children III/WISC-III), visual-motor integration (Beery VMI), and behavior (parent-completed Child Behavior Checklist and teacher-completed Teacher Report Form). The CANTAB tests executive function by challenging response speed and movement, short-term memory capacity, rapid recognition of patterns, spatial planning, rule acquisition, set shifting, and response inhibition. The BRIEF questionnaire, completed by parents and teachers, assesses behavioral regulation (inhibition of impulses, shifting of behavior in response to changing demands, and emotional control) and metacognition (task initiation, working memory, planning, organization, and monitoring of work and performance. Intelligence was assessed with the Wechsler Intelligence Scale for Children III (WISC-III), in which estimated full-scale IQ is highly predictive of later full-scale IQ.

There was no difference between the preemptive morphine (*n* = 43) and placebo (*n* = 46) groups in growth parameters, IQ scores, academic skills, visual-motor integration, or in the proportion of children attending mainstream education. Executive function as measured by the CANTAB did not differ between the groups, however the parent-scored BRIEF questionnaires showed that the preemptive morphine group had improved scores in the behavioral regulation (*p* = 0.006), metacognition (*p* = 0.03), and global executive composite indices (*p* = 0.009). Teacher-scored BRIEF questionnaires showed similar trends toward improved executive function in the preemptive morphine group, but these were not statistically significant. Behavioral scores were inconsistent, with the placebo group having more children with externalizing problems identified by parent questionnaire and the preemptive morphine group having more children with internalizing problems identified by teachers.

The morphine-associated adverse effects reported by the small NEOPAIN trial (lower body weight and head circumference, increased social problems, and poorer executive function as indicated by increased response latencies and lower task completion rates) were not observed in this larger study. Notably, the morphine infusion rate that these infants were exposed to (10 mcg/kg/h) was lower than the infusion rates used in the NEOPAIN trial (10–30 mcg/kg/h). Long-term neurologic sequelae related to morphine therapy may be dose-dependent.

### 5.3. A Study Entitled

Neonatal pain, parenting stress and interaction, in relation to cognitive and motor development at 8 and 18 months in preterm infants [31] investigated the cumulative effects of procedural pain and morphine exposure on neurodevelopmental outcome in a cohort of 137 very preterm infants. It also examined the modulating effects of parental stress and parent-infant interactions on the above. 74 full-term infants were used as a control cohort. Infants were assessed using the Bayley Scales of Infant Development II (Bayley II), the Mental Development Index (MDI), and the Psychomotor Development Index (PDI) at 18 and/or 18 months corrected gestational age. Parents were assessed with the Parenting Stress Index (PSI) and observed semi-structured teaching play.

Preterm infants had significantly lower MDI and PDI scores than full-term controls at 18 months (*p* = 0.02), and the parents of preterm infants had significantly higher PSI scores than the parents of their term counterparts at both 8 months (*p* = 0.04) and 18 months (*p* = 0.03). Several neonatal variables (number of skin breaking procedures, number of days of mechanical ventilation, lower gestational age, and increased morphine exposure) were significantly correlated with lower MDI and PDI. All of the above were statistically significant correlations at both 8 and 18 months, with the sole exception of morphine exposure, which was correlated with lower PDI but not MDI at 8 months. At 18 months, higher Parent Organization scores and higher maternal education were correlated with better MDI and PDI scores. Hierarchical regression analysis controlling for early illness severity, morphine exposure, and dexamethasone treatment showed that more skin-breaking procedures and more days on the ventilator predicted lower MDI at 8 and 18 months; higher parental education predicted higher MDI at 18 months. Similar analysis of PDI score predictors showed that, when controlled for early illness severity and dexamethasone exposure, both the number of skin-breaking procedures and greater morphine exposure were associated with lower PDI scores (morphine exposure only significant at 8 months), while a greater number of children at home was associated with higher PDI scores.

Overall, poor motor and cognitive function were associated with more severe illness, more painful procedures, greater duration of ventilation and greater morphine exposure. Although these variables may be viewed as mere surrogates for increased disease burden, their significant predictive value when controlling for early illness severity suggest that both exposure to painful procedures and exposure to morphine may play a role in the pathogenesis of adverse neurodevelopmental outcomes.

## 6. Summary

Pain management is increasingly recognized as a vital aspect of neonatal care as infants are subject to many painful interventions [1]. Acute pain can have negative short-term and long-term effects including deleterious acute physiologic responses and possible neuromodulation leading to altered responses to noxious stimuli [11,16,17,18,19,20,22]. These effects can be mitigated by provision of adequate analgesia, which may include both non-pharmacologic measures (e.g., swaddling) and opioid pharmacotherapy.

Despite the potential benefits of morphine administration in ameliorating both acute and chronic response to repeated painful stimuli in the preclinical model [6,53], there is also pre-clinical evidence suggesting possible harmful effects of neonatal morphine administration. Apoptosis has been noted in human neurons and microglia following morphine exposure [2] and behavioral changes have been noted in morphine-exposed animals [4,5]. These pre-clinical models are concerning, but provide only an incomplete model of the complex clinical environment of neonatal critical care, as the animals were exposed to morphine in the absence of noxious stimuli and lack the medical comorbidities associated with most NICU admissions.

Several clinical studies have attempted to examine the long-term developmental impact of neonatal morphine therapy (Supplementary Information, Table S1). Interestingly, these studies highlight some anomalies in certain social and motor activities but do not demonstrate marked changes in IQ or academic performance. These studies do not demonstrate severe long-term harm. However, given the complex nature of neonatal critical care and subsequent development, the true long-term impact of therapy may be difficult to quantify.

Based on available evidence, it is clear that morphine reduces the known harmful impact of pain but also acts on multiple developing neurologic pathways. There is no compelling evidence to support severe long-term harm, but subtler behavioral changes have been noted. Morphine use should continue to be based on clinical judgment, with treatment providers continuing to remain aware of an evolving body of evidence. Meanwhile, future studies should focus investigate the role for and long term effects of alternative analgesics such as acetaminophen and remifentanil. The limitations of the data on the long-term effects of neonatal morphine therapy may be seen as a reflection of the dilemma faced by all physicians caring for neonates, who must carefully weigh the benefits of acute interventions against the potential for long-term harm.

## Supplementary Files

Supplementary File 1 Supplementary Information (PDF, 77 KB)
