# Supplementary material for: The Neurodevelopmental Impact of Neonatal Morphine Administration
Source: Brain Sci. 2014 Apr 25;4(2):321–34. doi: 10.3390/brainsci4020321 (PMC4101480; doi:10.3390/brainsci4020321)
Supplement: Supplementary File 1 — Supplementary Information (PDF, 77 KB) [file brainsci-04-00321-s001.pdf]

Supplementary Information

Table S1. Several clinical studies have attempted to examine the long-term developmental impact of neonatal morphine therapy.

Studies of Long Term Clinical Outcomes of Neonatal Morphine Exposure	Result
Grunau *et al*, 2009 [31]—Neonatal pain, parenting stress and interaction, in relation to cognitive and motor development at 8 and 18 months in preterm infants.	Increasing amounts of Neonatal Morphine exposure correlated with worse motor outcome at 8 month but effect dissipated at 18 months
Ferguson *et al*, 2012 [45]—A pilot study of preemptive morphine analgesia in preterm neonates: effects on head circumference, social behavior, and response latencies in early childhood.	No significant difference in IQ or Academic Performance. However, parent reported social problems, increased response latencies, lower body weight and smaller head circumference were noted in the morphine treated group
De Graaf *et al*, 2013 [50]—Does neonatal morphine use affect neuropsychological outcomes at 8 to 9 years of age?	No evidence of negative impact of morphine infusion on IQ or executive function at 8 to 9 year follow-up. Possible Protective effect of infusion

© 2014 by the authors; licensee MDPI, Basel, Switzerland. This article is an open access article distributed under the terms and conditions of the Creative Commons Attribution license (http://creativecommons.org/licenses/by/3.0/).
